# The Inversion of SPAD Value in Pear Tree Leaves by Integrating Unmanned Aerial Vehicle Spectral Information and Textural Features

**DOI:** 10.3390/s25030618

**Published:** 2025-01-21

**Authors:** Ning Yan, Yasen Qin, Haotian Wang, Qi Wang, Fangyu Hu, Yuwei Wu, Xuedong Zhang, Xu Li

**Affiliations:** 1 College of Information Engineering, Tarim University, Alaer 843300, China; 18295549487@163.com (N.Y.); qys_1738828465@163.com (Y.Q.); 18537178053@163.com (H.W.); 15703411873@163.com (Q.W.); m15945632454@163.com (F.H.); 15770019308@163.com (Y.W.); 2 Key Laboratory of Tarim Oasis Agriculture, Ministry of Education, Tarim University, Alaer 843300, China

**Keywords:** pear tree leaf, SPAD, UAV multispectral, textural features, machine learning

## Abstract

Chlorophyll is crucial for pear tree growth and fruit quality. In order to integrate the unmanned aerial vehicle (UAV) multispectral vegetation indices and textural features to realize the estimation of the SPAD value of pear leaves, this study used the UAV multispectral remote sensing images and ground measurements to extract the vegetation indices and textural features, and analyze their correlation with the SPAD value of leaves during the fruit expansion period of the pear tree. Finally, four machine learning methods, namely XGBoost, random forest (RF), back-propagation neural network (BPNN), and optimized integration algorithm (OIA), were used to construct inversion models of the SPAD value of pear trees, with different feature inputs based on vegetation indices, textural features, and their combinations, respectively. Moreover, the differences among these models were compared. The results showed the following: (1) both vegetation indices and textural features were significantly correlated with SPAD values, which were important indicators for estimating the SPAD values of pear leaves; (2) combining vegetation indices and textural features significantly improved the accuracy of SPAD value estimation compared with a single feature type; (3) the four machine learning algorithms demonstrated good predictive ability, and the OIA model outperformed the single model, with the model based on the OIA inversion model combining vegetation indices and textural features having the best accuracy, with $R^{2}$ values of 0.931 and 0.877 for the training and validation sets, respectively. This study demonstrated the efficacy of integrating multiple models and features to accurately invert SPAD values, which, in turn, supported the refined management of pear orchards.

## 1. Introduction

Chlorophyll is the most important photosynthetic pigment in plant leaves, and its content is closely related to plant growth status, nutritional level, and stress resistance [1]. As one of the most important fruit trees in China, the monitoring of the chlorophyll content of pear leaves is of great significance in guiding the cultivation management and improving fruit quality, and the inversion of the SPAD value can be used to adjust the measures of fertilizer application, irrigation, and pest control in a timely manner [2]. The traditional method for measuring chlorophyll content has disadvantages such as cumbersome operation, destructiveness, poor timeliness, etc., making it difficult to meet the needs of large-scale and rapid monitoring [3]. At present, the measurement of relative leaf chlorophyll content can be realized by the SPAD-502Plus portable chlorophyll meter [4], which provides a high accuracy for single-crop measurement and can effectively reflect the health status of the crop and determine the optimal timing of fertilizer application. However, it is difficult for this method to meet the needs of large-scale crop production management.

The spectral properties of green vegetation in the visible and near-infrared ranges are influenced by the chlorophyll content, which exhibits different levels of absorption and reflection. Therefore, vegetation indices obtained from remotely sensed imagery are the key indicators for assessing the spatial distribution of crop chlorophyll content and its variability [5]. Remote sensing has been widely used to qualitatively and quantitatively analyze crop growth indicators in large-scale agricultural fields in a non-destructive manner [6]. However, the current free remote sensing data, such as MODIS series [7], Landsat series [8], and Sentinel-2 [9], have a low spatial resolution, which poses a challenge for conducting accurate agricultural field-scale studies [10]. In recent years, the development of UAV remote sensing technology has provided a new method for the rapid acquisition of plant physiological parameters, and UAV images are convenient for acquiring plant spectral images with their high spatial and temporal resolution, and flexible operation [11]. Many scholars have utilized UAV remote sensing [12] technology combined with machine learning to estimate crop chlorophyll content. Wang et al. [13] proposed a method based on a gradient boosting regression tree algorithm for inverting the chlorophyll content of winter wheat at different fertility stages, achieving high-precision inversion of this content. Tian et al. [14] extracted SPAD-sensitive features by using feature screening, adopted regression and machine learning methods for model construction, and finally obtained a SPAD inversion model of cotton with good generalization performance. Schirrmann et al. [15] applied RGB images acquired by UAV to study biological and chemical indicators such as relative chlorophyll content in wheat canopy, and established regression models between the content of each indicator and spectral indices. Yuan et al. [16] carried out the SPAD estimation of the slope barriers of tropical endangered tree species, and concluded that the random forest model had the highest prediction accuracy by comparing different algorithms.

Textural features are another important characteristic commonly used in remote sensing monitoring methods for crop growth. They are widely applied in estimating crop biomass, leaf area indices, and chlorophyll content, among other parameters [17]. Khosravi et al. [18] found that textural features, such as statistical characteristics, have the advantage of rotation invariance. This can compensate for the insensitivity of spectral information to the region size and orientation, and they demonstrate strong resistance to image noise. The application of textural information can significantly improve the accuracy of crop nutrition estimation. Zheng et al. [19] established an optimal nitrogen content estimation model for rice using multispectral vegetation indices and textural features, with the $R^{2}$ value reaching 0.91. Li et al. [20] confirmed that the models inputting parameters combining wavelet energy textural and spectral features based on images or gray-level co-occurrence matrix textural features combined with color indices have a higher accuracy in estimating rice LAI than models with single-parameter types. Studies have shown that the combination of textural features and vegetation indices can enhance the inversion accuracy of crop physiological growth indicators (biomass [21], leaf area indices [22], chlorophyll content [23], etc.). Constructing inversion models with this combined input provides a higher accuracy compared to single-input variables [24]. Therefore, this study further validated the inversion capability of models constructed with vegetation indices and textural information for the SPAD values of pear tree leaves.

In view of this, in this study, a variety of machine learning algorithms were used to construct inversion models for the SPAD value of pear tree leaves based on vegetation indices, textural features, and the combination of vegetation indices and textural features as inputs, respectively. The potential of different vegetation indices and textural features in estimating the SPAD value of pear tree leaves was explored, the optimal combination of vegetation indices and textural features was analyzed, and the model algorithm with the best performance in the estimation of the SPAD value of pear tree leaves based on multi-feature fusion was identified. This study enriches the research method system in this field. Through in-depth research on different vegetation indices, textural features, and their combinations, it provides a more comprehensive and accurate basis for feature selection in the nutritional diagnosis of pear trees. The determined optimal feature combination and model algorithm have high practical value and promotional significance. They are expected to provide new ideas and methods for the intelligent and precise development of pear tree planting management, and promote the in-depth application of agricultural remote sensing technology in the field of fruit tree cultivation.

## 2. Materials and Methods

### 2.1. Overview of the Study Area

**Figure 1:**
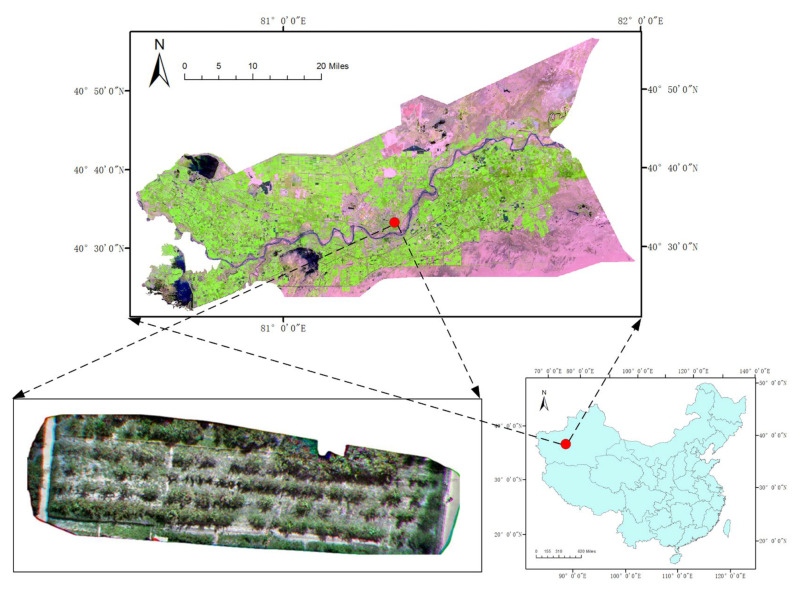
Overview map of the study area.

This study selected the horticultural experimental base of Tarim University in Alar City, Xinjiang, as the core research area (81°17′57″ E, 40°32′27″ N). This region is located in the northwest corner of Alar City, Xinjiang Uygur Autonomous Region, and enjoys a unique warm temperate continental arid climate [25]. This zone has distinctive climatic characteristics, with cold and dry winters and scorching summers. This provides unique natural conditions for the study of the growth adaptation and chlorophyll dynamics of horticultural crops under extreme climatic conditions, and the test area is flat and free of obstructions, providing good conditions for UAV operations. The experiment was chosen to be conducted in early July 2024 (fruit expansion period), when leaf chlorophyll almost reached its peak and growth conditions were representative. Sixty Korla Fragrant Pear trees in the designed experimental area were used as samples for the experiment. The schematic diagram of the study area is shown in Figure 1.

### 2.2. Data Acquisition and Processing

#### 2.2.1. UAV Image Acquisition and Pre-Processing

In this study, we used a DJI Phantom 4 multispectral version of the UAV. It employs a centimeter-level positioning system and synchronizes the flight control, the camera, and the clock system of real-time kinematic (RTK) with microsecond-level precision. The UAV was equipped with a multispectral sensor, which integrates one visible camera and five multispectral cameras (blue, green, red, red edge, and near infrared). The center wavelength of the blue band was 450 nm and the bandwidth was 32 nm. The center wavelength of the green band was 560 nm and the bandwidth was 32 nm. The center wavelength of the red band was 650 nm and the bandwidth was 32 nm. The center wavelength of the red edge band was 730 nm and the bandwidth was 32 nm. The center wavelength of the NIR band was 840 nm and the bandwidth was 52 nm. Meanwhile, the UAV has a built-in GPS and IMU system. To ensure the consistency of light conditions at the moment of data collection, the multispectral image data from the UAV were collected during the fruit expansion period (4 July 2024) of the pear tree on a windless and sunny day (12:00 p.m. to 2:00 p.m.). Before takeoff, the UAV was manually controlled to fly to about 2.5 m directly above the calibrated whiteboard and the camera was used in the single-shot mode to photograph the standard whiteboard. The flight mode of the UAV followed the pre-planned route. The heading overlap degree was set to 80%, the side overlap degree was 70%, the UAV flight height was 12 m, the flight speed was 3.5 m/s, the sensor lens was vertically downward (the pitch angle was 0 degrees), and the photo-taking mode was an isochronous time-interval mode with a time interval of 3 s. The image resolution was 1600 pixels × 1300 pixels, and the ground resolution was 5 cm. To prevent geometric distortion at the edges of the orthophotos after the splicing of the sample plots, the photographic area was extended outward during the route planning.

The UAV multispectral image preprocessing includes image stitching, geometric correction, radiometric correction, and reflectance extraction. First, the acquired images were spliced into a complete visible image of the study area and five single-band orthophotos using Pix4Dmapper 4.5.6 software. Then, a multispectral image with six bands was obtained by band synthesis using ENVI 5.6 software. Next, geometric correction was performed using ENVI 5.6 software to ensure that the correction error was within 0.5 image pixel. Subsequently, the whiteboard correction method was used for radiometric correction to convert DN values into spectral reflectance values. Finally, the reflectance of the sample area was extracted by performing region-of-interest interception and manually sketching the images of some pear tree leaves in ArcMap 10.8 software for subsequent vegetation index construction.

#### 2.2.2. Ground Truthing Data Acquisition

The SPAD-502 portable chlorophyll meter was used to collect SPAD values from pear tree leaves. The equipment used in the experiment was the SPAD-502 portable chlorophyll meter. For each pear tree, 10 healthy and mature leaves were randomly selected from the middle and upper parts of the canopy to measure their SPAD values. Five measurements were taken at different positions on each leaf, and the average value was calculated. The average SPAD value of the 10 leaves was then determined as the SPAD value for that pear tree. To ensure reliability, the experiment was repeated three times. Ground measurements of the SPAD values of pear tree leaves were conducted in synchronization with the UAV flights. A total of 60 SPAD sample data points were obtained from pear tree leaves, with a maximum value of 44.94, a minimum value of 35.26, a mean value of 40.78, a standard deviation of 1.92, and a coefficient of variation of 0.05.

#### 2.2.3. Multispectral Vegetation Index Selection and Calculation

The vegetation spectral index (VSI) is a type of specific remote sensing index that assesses the condition and health of vegetation by analyzing its spectral reflectance characteristics. These indices are calculated based on the ratios of the light reflected or absorbed by vegetation after interacting with different wavelengths of light, such as visible, near-infrared, and shortwave-infrared light [26]. Following related research, this study selected 11 vegetation indices for correlation analysis with the SPAD values of pear tree leaves. The selected vegetation indices included NDVI, GNDVI, RVI, GRVI, DVI, GBNDVI, OSAVI, CIgreen, SAVI, SIPI, and EVI. Subsequently, using the spectral data extracted from 60 sampling points in the multispectral imagery, the calculation of vegetation indices was performed in the Python 3.10 environment according to the formulas presented in Table 1.

#### 2.2.4. Multispectral Textural Feature Extraction

Texture refers to the presence of similar patterns with strong or weak regularity within an image. It is a common visual phenomenon characterized by the recurring local structures or arrangement rules in the image [27]. Among various textural feature analysis methods, the gray-level co-occurrence matrix (GLCM) is a commonly used technique for quantifying the textural information of an image.

This study employed ENVI 5.6 software to extract textural features from images using second-order statistical methods, specifically co-occurrence measures. A total of eight textural feature values were obtained from the five multispectral bands (as shown in Table 2): mean (mean), variance (var), homogeneity (hom), contrast (con), dissimilarity (dis), entropy (ent), second moment (sem), and correlation (cor). Considering the spatial resolution of the imagery and the actual arrangement characteristics of fruit trees, a 3×3 window size and a 45° angular direction were adopted during the extraction process. Additionally, the offsets for the spatial correlation matrices, X and Y, were both set to their default values of 1. With this configuration, a total of 40 pieces of textural feature information were acquired, providing robust support for subsequent research.

### 2.3. Model Construction and Evaluation

#### 2.3.1. Model Construction Method

A total of 60 valid samples were collected in this experiment. When establishing the inversion model for the SPAD values of pear tree leaves, 70% of the valid sample data were randomly selected as the training set, and the remaining 30% were used as the validation set. XGBoost, RF, BPNN, and OAI were, respectively, employed to construct the inversion models for the SPAD values of pear tree leaves, with three input modes, including vegetation indices, textural features, and the combination of vegetation indices and textural features.

(1) eXtreme Gradient Boosting Algorithm

The eXtreme Gradient Boosting Algorithm, abbreviated as XGBoost, is an efficient machine learning model based on gradient boosting decision trees [28]. The model adopts the core idea of gradient boosting trees: each tree is built on the residuals of the previous tree, and the errors of the previous tree are corrected through continuous iteration, which ultimately results in a powerful integrated model. To prevent overfitting, XGBoost employs regularization techniques, including L1 and L2 regularization terms, learning rate reduction, and column sampling, which makes the model more generalizable and less susceptible to noise and complexity in the training data. XGBoost, as a Boosting algorithm, contains three elements of Boosting: loss function to measure the difference between the predicted results of the model and the true value; weak evaluators, generally decision trees (CART and DART trees), based on the different Boosting algorithms using different tree building processes; and an integration method to combine the results, that is, the integration algorithm specific output integration results. The objective function is defined as

(1) $$Obj=\sum_{i=1}^{n}l\left(y_{i}^{\prime },\;y_{i}\right)+\sum_{k=1}^{k}\Omega \left(f_{k}\right)$$

where $y_{i}^{\prime }$ represents the predicted value of sample *i*; $y_{i}$ represents the true value of sample *i*; $l\left(y_{i}^{\prime },y_{i}\right)$ represents the loss function; $\Omega \left(f_{k}\right)$ represents the regularization term; *n* represents the total number of samples; and *k* represents the number of samples of the regularization term.

In this study, the XGBoost algorithm was selected mainly because its implementation enables the algorithm to cache data and utilize multiple CPU cores for rapid processing. This improves the training speed and efficiency. Moreover, it performs excellently on large-scale datasets. Compared with traditional decision tree construction methods, XGBoost can provide an evaluation of the importance of each feature, which is helpful for understanding the prediction and decision-making process of the model.

(2) Random Forest Algorithm

The random forest (RF) algorithm is an integrated learning method based on decision trees [29]. The core idea was to introduce randomness so that each tree can learn different patterns from different feature subsets, thus forming a diverse and generalization-capable model. In prediction, RF uses majority voting (classification problem) or averaging (regression problem) to integrate the prediction results of all trees. This integration strategy effectively reduces the variance of the prediction and improves the accuracy of the model, the RF algorithm is not only suitable for classification and regression tasks, but also can be used for anomaly detection and feature selection in unsupervised learning. The prediction of the RF algorithm can be expressed as

(2) $$\hat{Y}=\frac{1}{N_{t}}\sum_{i=1}^{N_{t}}f_{i}\left(x\right)\;$$

where $\hat{Y}$ is the final prediction, $N_{t}$ is the number of decision trees, and $f_{i}\left(x\right)$ is the prediction of input sample X by the ith decision tree. The RF algorithm is an efficient, robust, and easy-to-implement machine learning model, which has been widely used in the field of data analytics and machine learning because it improves the generalization ability of the model by introducing randomness while maintaining high prediction accuracy. The selection of the RF algorithm in this study is mainly attributed to its excellent prediction performance, efficient data processing capability, and unique feature importance assessment ability, making it highly accurate and robust.

(3) Back-Propagation Neural Network Algorithm

The back-propagation neural network (BPNN) is one of the most widely used algorithms in artificial neural network algorithms [30]. Its core lies in adjusting the connection weights and bias parameters of the network to minimize the prediction error. During the training stage of the network, first, through the forward propagation process, the network generates predicted outputs using the input data. Subsequently, the errors between the predicted values and the actual values are calculated. Based on these errors, the network conducts back propagation through optimization techniques such as gradient descent to correct the weights and biases, thereby reducing the errors. This iterative cycle will continue until specific termination conditions are met, for example, when the error drops below a predetermined level or the number of iterations reaches a predetermined maximum. Its input variables, output variables, activation functions, and empirical formulas are as follows:

(3) $$\;{net}_{i}=\sum_{j=1}(w_{ij}.x_{j}+b$$

(4) $$\;f\left(x\right)=\frac{1}{{1+e}^{-x}}$$

(5) $${\;y}_{i}=f\left({net}_{i}\right)$$

(6) $$\;s=\sqrt{0.43mn+0.12nn+2.54m+0.77n+0.35}+0.5$$

Equation (3) denotes the input variable ${net}_{i}$ of the ith node of the hidden layer of the neural network, $x_{j}$ denotes the input variable of the jth node of the hidden layer, and $w_{ij}$ denotes the connection weight from the ith node of the hidden layer to the jth node of the input layer. Equation (4) denotes the activation function, Equation (5) denotes the output variable of the ith node of the hidden layer, and the empirical formula used for the number of neurons in the hidden layer in Equation (6), in which b represents the threshold parameter, and m and n represent the number of nodes in the input and output layers, respectively.

In this study, the BPNN-based inversion model for the SPAD values of pear tree leaves was established. In the process of model construction with vegetation indices, textural features, and the combination of vegetation indices and textural features as inputs, the structure of the input layer–hidden layer–output layer was set as 6–15–1. The target error threshold was set to 10^−13^, the learning rate was 0.01, and the maximum number of iterations was 1000.

(4) Optimization Integration Algorithm Model

The optimized integration algorithm model, abbreviated as OIA, is an efficient machine learning technique. Its basic concept is to ingeniously integrate multiple base models to construct a prediction model with better performance and stronger generalization ability. The key to this technique lies in taking advantage of the mutual complementarity among different models and improving the overall prediction accuracy through different integration methods [31]. The OIA model focuses on the differences among base models and effectively integrates the prediction results of each model through various integration strategies to enhance the prediction effect. In addition, the OIA model also attaches importance to the optimization of base models, including parameter tuning, feature selection and so on, aiming to improve the performance of individual models. Eventually, this integration method not only reduces the risk of overfitting, but also significantly enhances the model’s generalization ability on unknown data, making it possess stronger predictive power and reliability in complex and variable data environments. In practical applications, the OIA model has become a powerful tool for solving various machine learning problems. In particular, when dealing with high-dimensional and nonlinear problems, its performance is often superior to that of a single model.

**Figure 2:**
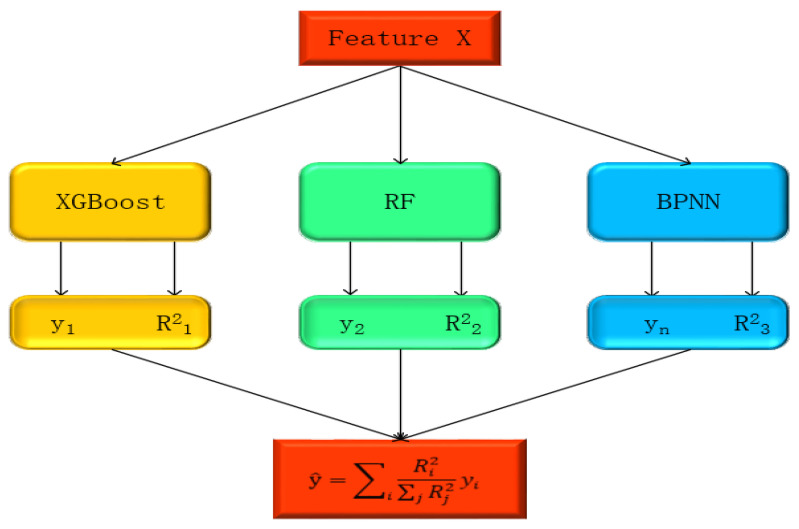
Optimization integration algorithm model diagram.

The integration method adopted in this study is linear fusion. As shown in Figure 2, the prediction results of the three individual models, namely XGBoost, RF, and BPNN, are weighted. The weight of each model is determined according to its score on the validation set. By normalizing the scores of the XGBoost, RF, and BPNN models, and then using the normalized scores as weights to conduct linear weighting on the prediction results of these three models, the final prediction result can be obtained, thus achieving accurate estimation of the SPAD values of pear tree leaves.

#### 2.3.2. Evaluation of Model Accuracy

In order to verify the model prediction accuracy and prediction ability, the determination coefficient ($R^{2}$), root mean square error (RMSE), and relative percent difference (RPD) were selected to evaluate the model accuracy [32]. The larger the $R^{2}$ and the smaller the RMSE of the estimation and validation models, the better the stability of the model and the higher the prediction accuracy; the RPD value between 1.4 and 2.0 indicates that the model has a general level of prediction, and when the RPD value is greater than 2.0, it indicates that the model has a high prediction accuracy. The specific formulas are shown in Equations (7)–(9).

(7) $$R^{2}=\frac{\sum_{i=1}^{n}{\left(f_{i}-y_{i}\right)}^{2}}{\sum_{i=1}^{n}{\left(f_{i}-{\bar{f}}_{i}\right)}^{2}}$$

(8) $$RMSE=\sqrt{\frac{\sum_{i=1}^{n}({f_{i}-y_{i})}^{2}}{n}}$$

(9) $$RPD=\frac{{SD}_{fi}}{RMSE}$$

In the formula,$f_{i}$ and $y_{i}$ denote the true and predicted values of the ith sample target, respectively, ${\bar{f}}_{i}$ denotes the average of the true values of all sample targets in the sample set, n denotes the number of samples, and ${SD}_{fi}$ denotes the standard deviation of $f_{i}$.

We used the Pearson correlation coefficient to assess the correlation between the SPAD values of pear tree leaves and vegetation indices, as well as textural features, denoted by r, with a range of [−1,1]; the calculation formula can be found in Equation (10).

(10) $$r=\frac{{\left[\sum_{i=1}^{n}\left(x_{i}-\bar{x}\right)\left(y_{i}-\bar{y}\right)\right]}^{2}}{\sum_{i=1}^{n}{\left(x_{i}-\bar{x}\right)}^{2}\sum_{i=1}^{n}{\left(y_{i}-\bar{y}\right)}^{2\;}}$$

In the formula, $x_{i}$ and $y_{i}$ represent the observed values of the two variables, while $\bar{x}$ and $\bar{y}$ denote the mean values of these two variables, respectively.

The closer the absolute value of the correlation coefficient (r) is to 1, the stronger the correlation between the variables. As shown in Table 3, vegetation indices with absolute values of correlation coefficients greater than 0.6 and textural features with absolute values of correlation coefficients greater than 0.4 were selected for modeling in this study.

## 3. Results

### 3.1. Vegetation Index Correlation Analysis and Selection

**Figure 3:**
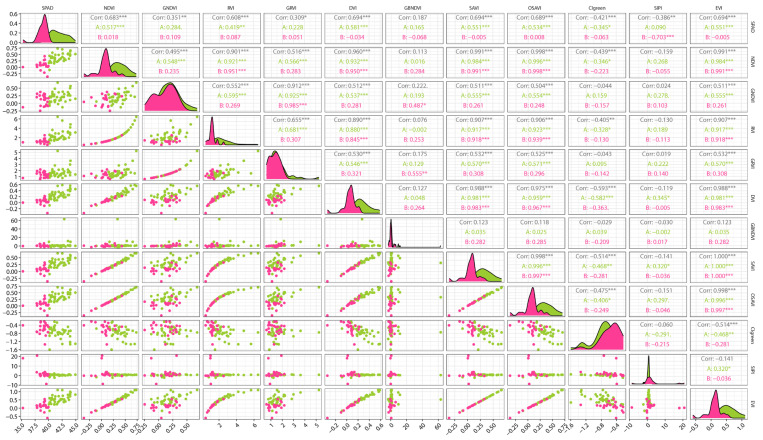
Vegetation indices and SPAD correlation analysis. (Note: Corr denotes the overall SPAD correlation, A represents the correlation when SPAD > 40, and B represents the correlation when SPAD < 40. The green dots represent samples with SPAD > 40, indicating that the pear trees have a relatively high chlorophyll content. The pink dots represent samples with SPAD < 40, indicating that the chlorophyll content of the pear trees is relatively low. * indicates a significant correlation at the 0.05 significance level, ** indicates a significant correlation at the 0.01 significance level, and *** indicates a significant correlation at the 0.001 significance level).

Pearson correlation coefficient analysis was conducted on the selected 11 vegetation indices and the SPAD values of pear tree leaves. The results of the correlation analysis are shown in Figure 3. Among the 11 vegetation indices, CIgreen and SIPI exhibited a negative correlation with SPAD, while the remaining 9 vegetation indices showed a positive correlation with SPAD. The correlation coefficients between NDVI, OSAVI, SAVI, EVI, RVI, and DVI and SPAD ranged from 0.608 to 0.694, and all were significantly correlated at the 0.01 level. There was a weak correlation between GNDVI, CIgreen, SIPI, and GRVI and SPAD. The correlation coefficient of GBNDVI was 0.187, indicating no significant correlation with SPAD. During the fruit expansion period of pear trees in early July 2024, the optimal correlation coefficient of the correlation degree between DVI, EVI, SAVI, and the SPAD values of pear tree leaves reached 0.694. The correlation coefficients of OSAVI, NDVI, and RVI reached 0.689, 0.683, and 0.608 respectively. These six vegetation indices all had a relatively strong correlation with the SPAD values of pear tree leaves. Therefore, it is feasible to select these six vegetation indices, namely DVI, EVI, SAVI, OSAVI, NDVI, and RVI, to estimate the SPAD values of pear tree leaves.

### 3.2. Textural Feature Correlation Analysis and Selection

In this study, according to Section 2.2.4, Pearson correlation analysis was conducted on all the extracted multispectral textural features and the obtained SPAD values of pear tree leaves. The results are shown in Table 4. The combination of row (Y) and column (X) represents the textural feature value of Y in the X band. For example, R-mean represents the mean value in the red light band. As can be seen from Table 4, the correlation coefficients between R-mean, B-mean, R-dis, R-contrast, G-mean, R-var, and the SPAD values of pear tree leaves ranged from 0.410 to 0.590, showing a relatively good correlation and were significantly correlated at the level of *p* < 0.01. Thirteen textural feature information, including R-hom, R-entropy, R-sec, G-var, G-hom, G-contrast, G-dis, G-entropy, B-var, B-hom, B-contrast, B-dis, and B-entropy had a general correlation with the SPAD values of pear tree leaves, while the correlation between the remaining textural features and the SPAD values of pear tree leaves were not obvious. Therefore, it is feasible to select these six textural features, namely R-mean, B-mean, R-dis, R-contrast, G-mean, and R-var, to estimate the SPAD values of pear tree leaves.

### 3.3. The Best Model for Predicting SPAD in Pear Leaves

#### 3.3.1. The Best Model Based on Vegetation Indices

**Figure 4:**
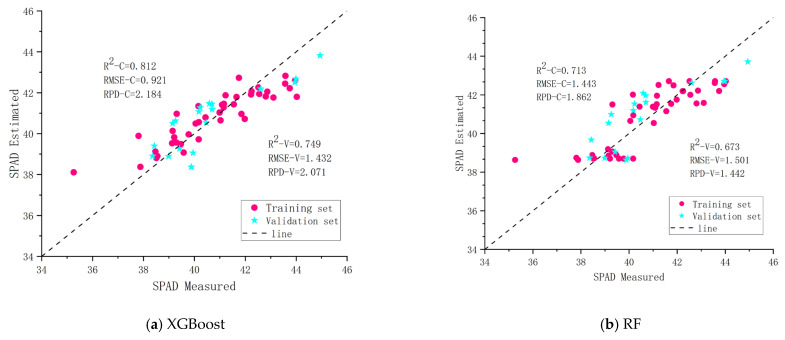

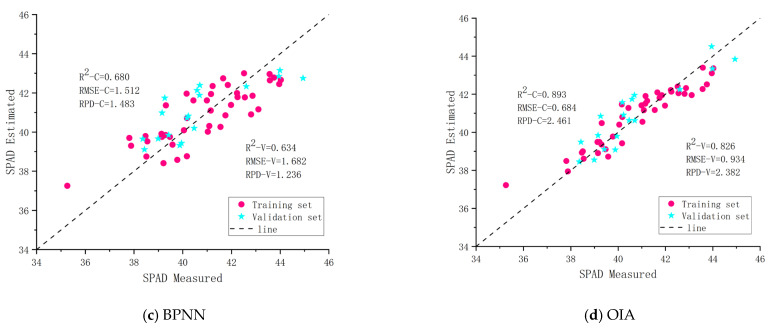
SPAD inversion results of different algorithms based on vegetation indices: (**a**) XGBoost; (**b**) RF; (**c**) BPNN; (**d**) OIA.

This study selected the six vegetation indices with the highest correlation coefficients (DVI, EVI, SAVI, OSAVI, NDVI, and RVI) as independent variables and the SPAD values of pear tree leaves as the dependent variable, based on the analysis of the correlation between vegetation indices and the SPAD values of pear tree leaves (as shown in Figure 4). XGBoost, RF, BPNN, and OIA were employed to construct the inversion models for the SPAD values of pear tree leaves. Additionally, the estimation accuracy of the models ($R^{2}$, RMSE, RPD) was assessed by comparing the predicted values with the measured values. As depicted in Figure 4, the $R^{2}$ values for the training set across the four modeling methods ranged from 0.680 to 0.893, and for the validation set from 0.634 to 0.826. The results indicate that among the four modeling methods, OIA performed the best (with $R^{2}$ = 0.893, RMSE = 0.684, RPD = 2.461 for the training set; with $R^{2}$ = 0.826, RMSE = 0.934, RPD = 2.382 for the validation set), while the BPNN model had the lowest accuracy (with $R^{2}$ = 0.680, RMSE = 1.512, RPD = 1.483 for the training set; with $R^{2}$ = 0.634, RMSE = 1.682, RPD = 1.236 for the validation set). Therefore, based on vegetation indices, the OIA model achieved the highest accuracy for constructing the SPAD value inversion model of pear tree leaves.

#### 3.3.2. The Best Model Based on Textural Features

**Figure 5:**
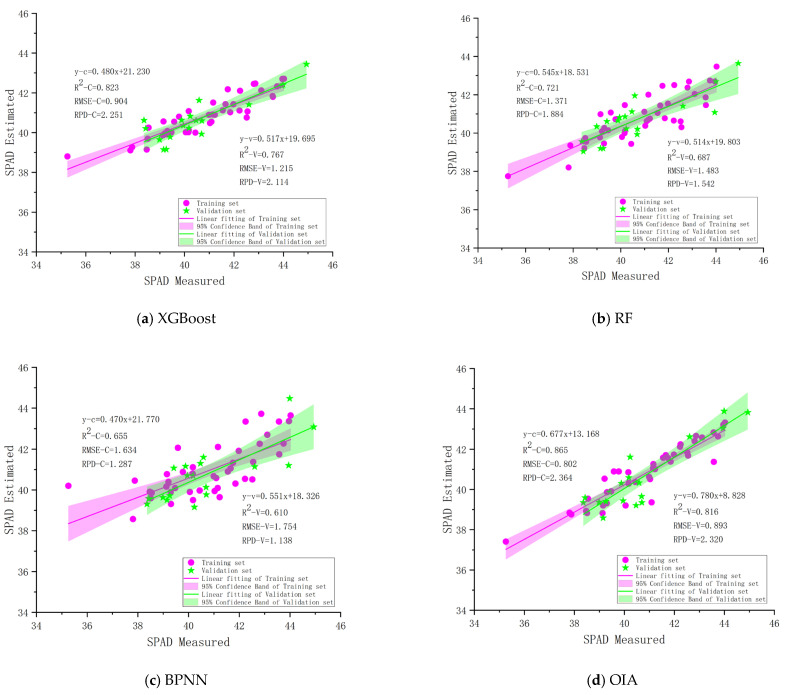
SPAD inversion results of different algorithms based on textural features: (**a**) XGBoost; (**b**) RF; (**c**) BPNN; (**d**) OIA.

This study selected the six textural features with the highest correlation coefficients (R-mean, B-mean, R-dis, R-contrast, G-mean, and R-var) as independent variables and the SPAD values of pear tree leaves as the dependent variable, based on the analysis of the correlation between textural features and the SPAD values of pear tree leaves (as shown in Table 4). XGBoost, RF, BPNN, and OIA were employed to construct the inversion models for the SPAD values of pear tree leaves. Additionally, the estimation accuracy of the models ($R^{2}$, RMSE, RPD) was assessed by comparing the predicted values with the measured values. As depicted in Figure 5, in terms of model algorithms, the $R^{2}$ values for the training set across the four modeling algorithms ranged from 0.655 to 0.865, and for the validation set from 0.610 to 0.816. The BPNN model had the lowest accuracy (with $R^{2}$= 0.655, RMSE = 1.634, RPD = 1.287 for the training set; with $R^{2}$= 0.610, RMSE = 1.754, RPD = 1.138 for the validation set), followed by the RF model (with $R^{2}$= 0.721, RMSE = 1.371, RPD = 1.884 for the training set; with $R^{2}$= 0.687, RMSE = 1.483, RPD = 1.542 for the validation set). XGBoost and OIA models exhibited better accuracy, with training set $R^{2}$ values above 0.8 and validation set $R^{2}$ values above 0.7. Therefore, among the inversion models for the SPAD values of pear tree leaves based on the selected textural features, the XGBoost and OIA models achieved the highest accuracy.

#### 3.3.3. The Best Model for Combining Vegetation Indices with Textural Features

**Figure 6:**
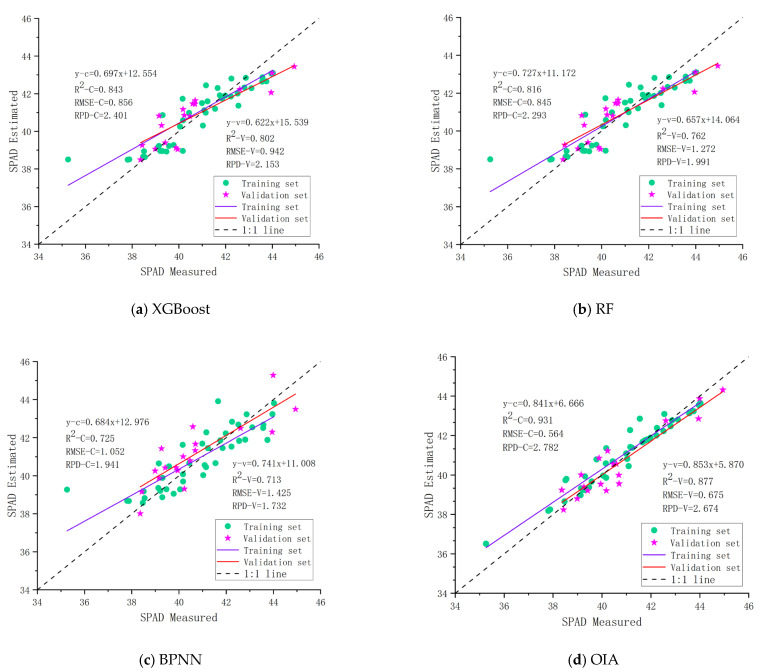
SPAD inversion results of different algorithms based on combining vegetation indices and textural features: (**a**) XGBoost; (**b**) RF; (**c**) BPNN; (**d**) OIA.

To explore the impact of different modeling approaches on the SPAD values of pear tree leaves and to enhance the estimation accuracy of the models, a combination of vegetation indices and textural features was used as input parameters for XGBoost, RF, BPNN, and OIA to construct the inversion models for the SPAD values of pear tree leaves. As shown in Figure 6, the accuracy of the SPAD value estimation models improved for all four modeling methods after the addition of textural features, with the OIA method performing most prominently (with $R^{2}$= 0.931, RMSE = 0.564, RPD = 2.782 for the training set; with $R^{2}$= 0.877, RMSE = 0.675, RPD = 2.674 for the validation set). Compared to the OIA model constructed based solely on vegetation indices (for the training set: $R^{2}$ increased by 0.038, RMSE decreased by 0.120, RPD increased by 0.321; for the validation set: $R^{2}$ increased by 0.051, RMSE decreased by 0.259, RPD increased by 0.292), and the model based solely on textural features (for the training set: $R^{2}$ increased by 0.066, RMSE decreased by 0.238, RPD increased by 0.418; for the validation set: $R^{2}$ increased by 0.061, RMSE decreased by 0.218, RPD increased by 0.354), the OIA model with vegetation indices and textural features as independent variables achieved the highest prediction accuracy. The slope of the fitting line of this model is very close to 1, indicating that the inversion capability of the model is relatively stable and represents an effective method for improving the inversion accuracy of SPAD values.

### 3.4. Model Evaluation

The prediction accuracies of the four machine learning methods were compared. Inversion models were established with three input modes, respectively, namely based on vegetation indices, textural features, and the combination of vegetation indices and textural features. The statistical comparison of the model accuracy parameters is shown in Table 5. For BPNN and OIA, in the inversion models with vegetation indices as independent variables, both $R^{2}$ and RPD were higher than those of the models with textural features as the independent variables, while the RMSE was lower than that of the models with textural features as independent variables. For XGBoost and RF, in the inversion models with textural features as independent variables, both $R^{2}$ and RPD were higher than those of the models with vegetation indices as independent variables, and the RMSE was lower than that of the models with vegetation indices as independent variables. Compared with the models using only vegetation indices or textural features, the accuracies of the four models with the combination of vegetation indices and textural features as independent variables were all improved. This may be because the models based on vegetation indices could be limited under certain circumstances. For example, under high vegetation coverage or in the presence of shadows of ground objects, the results of vegetation indices might be affected. The combination of vegetation indices and textural features offers a more comprehensive feature description, providing richer information. Through research and analysis, it was found that among the four machine learning methods, the OIA was the optimal one for inverting the SPAD values of pear tree leaves. For the model with vegetation indices as independent variables, the $R^{2}$ values of the training set and the validation set were 0.893 and 0.826, respectively, the RMSE values were 0.684 and 0.934, respectively, and the RPD values were 2.461 and 2.382, respectively. For the model with textural features as independent variables, the $R^{2}$ values of the training set and the validation set were 0.865 and 0.816, respectively, the RMSE values were 0.802 and 0.893, respectively, and the RPD values were 2.364 and 2.320, respectively. For the model with the combination of vegetation indices and textural features as independent variables, the $R^{2}$ values of the training set and the validation set were 0.931 and 0.877, respectively, the RMSE values were 0.564 and 0.675, respectively, and the RPD values were 2.782 and 2.674, respectively. It can be seen that using the OIA method to construct the inversion model for the SPAD values of pear tree leaves based on the combination of vegetation indices and textural features has relatively good estimation accuracy. The inversion ability of this model is relatively stable and it is a relatively effective method to improve the inversion accuracy of SPAD values.

## 4. Discussion

Chlorophyll content is a key indicator of crop photosynthesis and growth, which is important for guiding the precise application of nitrogen fertilizer in agricultural fields [33]. The traditional field sampling and ground remote sensing techniques have the problems of high cost and lack of precision in obtaining the spatial distribution of crop chlorophyll. In recent years, some progress has been made in researching the chlorophyll content of potato, wheat, cotton, and maize using UAV remote sensing techniques, but these studies mainly rely on vegetation indices to estimate the chlorophyll content of crops, and the study of combining UAV multispectral vegetation indices with textural features and applying them to the estimation of SPAD in pear leaves is yet to be explored in depth.

Firstly, this study analyzed the correlation of different spectral vegetation indices and textural features with the SPAD values of pear leaves. Similar to the results of previous studies, the vegetation indices and textural features extracted based on UAV multispectral imagery had a better correlation with the SPAD values of pear leaves. In this paper, we obtained the correlation coefficients of NDVI, OSAVI, SAVI, and EVI through Pearson correlation analysis. The correlation coefficients between NDVI, OSAVI, SAVI, EVI, RVI, DVI, and SPAD were high, in the range of 0.608–0.694, which indicated that these six vegetation indices were sensitive to the SPAD of pear leaves; and among them, the correlation between NDVI, SAVI, and OSAVI was the highest, which was similar to the results of Wang et al. [34], who utilized the combination of the feature selection strategy of the UAV multispectral image and the feature fusion strategy for the estimation of the SPAD of winter wheat estimation. The textural features R-mean, B-mean, R-dis, R-contrast, G-mean, and R-var had a good correlation with pear leaf SPAD values, in the range of 0.410 to 0.590; however, differing from previous research results, this study showed that the red-edge band correlation vegetation indices and their textural features did not show optimal performance in estimating crop chlorophyll content. On the contrary, in the studies in the literature [35,36,37], the red-edge band and its indices maintained high sensitivity at high biomass levels, which significantly improved the accuracy of crop chlorophyll content estimation. This may be related to the vegetation types and environmental conditions in different regions. Based on the above correlation analysis, in terms of modeling, the SPAD inversion models of pear leaves based on vegetation indices, textural features, and the combination of vegetation indices and textural features as inputs were constructed by combining four machine learning methods with a higher accuracy than that of vegetation indices and textural features only, which may be attributed to the fact that the combination of vegetation indices and textural features provides richer and more diversified information, and the combination of both can provide more comprehensive characterization compared with the use of vegetation indices or textural features only. This may be because combining vegetation indices and textural features provides richer and more diversified information than using only vegetation indices or textural features, and the combination of these two features provides a more comprehensive characterization, with vegetation indices usually reflecting the growth status and photosynthetic activity of the vegetation [38], and textural features providing information on the structure of the vegetation canopy, as well as its spatial layout [39].The optimized integrated algorithm model has a combination effect that is generally better than the inversion results of a single model. The UAV multispectral vegetation indices and textural features simultaneously as the input features of the regression algorithm can effectively improve the performance of the estimation of the SPAD value of pear leaves. And compared with the models of XGBoost, RF, and BPNN, the $R^{2}$ of the validation set of the optimized integrated algorithm model increased by 0.075, 0.115, and 0.164, respectively. The RMSE was decreased by 0.267, 0.597, and 0.750, and RPD was increased by 0.521, 0.683, and 0.942, respectively. At the same time, similar to the previous study [40], the optimized integrated algorithm model has a better performance for estimating chlorophyll content. The model in this study has demonstrated good estimation performance in small-scale areas, but its application effectiveness in large-scale regions (such as county and city levels) requires further validation. The collection of multi-spectral images over extensive farmland using UAV is time-consuming and labor-intensive, and it is practically challenging. In this context, satellite data combined with UAV remote sensing monitoring can be utilized. PENG et al. [41] successfully inverted the SPAD values of soybean and corn in Nebraska, USA, by integrating Sentinel-2A MSI satellite imagery with ground-based hyperspectral measurement data. Due to the differences in spectral bands between satellite and UAV imagery, the scaling from UAV to satellite levels is an area that necessitates further research. Overall, the study results indicate that the fusion of vegetation indices with textural features is significant for estimating the SPAD values of pear tree leaves. However, there are some limitations in the study, such as the large number of extracted textural features, which poses a higher challenge for the model’s fitting ability, making the search for a more effective model even more challenging. Moreover, the correlation between most individual textural features and chlorophyll content is not significant. Future research could attempt to normalize textural features using methods similar to those used in constructing vegetation indices to explore whether their correlation can be significantly enhanced.

In summary, the combination of vegetation indices and textural features extracted from UAV multispectral images and the use of machine learning regression techniques can effectively estimate the SPAD value of pear tree leaves, thus providing the necessary information support for the fine management of the field.

## 5. Conclusions

This study explored the inversion of the SPAD values of pear tree leaves by UAV multispectral vegetation indices and textural features. Four machine learning methods were used to construct three precise inversion models of the SPAD values of pear tree leaves based on vegetation indices, textural features, and the combination of vegetation indices and textural features, respectively. The following conclusions were drawn: (1) among the 11 vegetation indices selected in this study, NDVI, OSAVI, SAVI, EVI, RVI, and DVI had good correlations with SPAD, with correlation coefficients ranging from 0.608 to 0.694. Among the 40 extracted textural features, R-mean, B-mean, R-dis, R-contrast, G-mean, and R-var had good correlations with the SPAD values of pear tree leaves, with correlation coefficients ranging from 0.410 to 0.590. This indicates that these six vegetation indices and textural features are relatively sensitive to the SPAD values of pear tree leaves, thus demonstrating that vegetation indices and textural features are important indicators for estimating the SPAD values of pear tree leaves. (2) Through comparison, it was found that the inversion model of the SPAD values of pear tree leaves with the input of the combination of vegetation indices and textural features had a better performance, and its prediction accuracy was higher than that of the models with single vegetation indices or textural features. This may be because the vegetation index model is limited under circumstances such as shadows, while the combined model integrates multiple vegetation indices and texture information reflecting the surface characteristics of crops, which can largely weaken the above deficiencies and better invert the SPAD values of pear tree leaves. (3) In this study, the accuracy of the inversion results of different models generally improved, indicating that the selected models had a certain degree of efficiency and reliability in inverting the SPAD values of pear tree leaves. In addition, the performance of the optimized integrated algorithm model usually surpassed that of a single model, which suggests that by integrating multiple models, their respective advantages can be more effectively utilized, thereby improving the accuracy and stability of the inversion results. Through experimental data and analysis in this study, it was concluded that the optimized integrated algorithm model combining XGBoost, RF, and BPNN had obvious advantages in inverting the SPAD values of pear tree leaves. This finding provides valuable references and guidance for future research and practice in related fields.

## Figures and Tables

**Table 1:** UAV multispectral vegetation indices and calculation formula.

Vegetation Index	Calculation Formula
NDVI	NDVI=(NIR−Red)/(NIR+Red)
GNDVI	GNDVI=(NIR−Green)/(NIR+Green)
RVI	RVI=NIR/Red
GRVI	GRVI=NIR/Green
DVI	DVI=NIR−Red
GBNDVI	GBNDVI=(NIR−Green)/(NIR+Green−2∗Blue)
OSAVI	OSAVI=1.16∗(NIR−Red)/(NIR+Red+0.16)
CIgreen	CIgreen=NIR/(Green−1)
SAVI	$SAVI=\left(1+L\right)*(NIR-Red)/(NIR+Red+L)$
SIPI	SIPI=(NIR−Blue)/(NIR−Red)
EVI	EVI=2.5∗(NIR−Red)/(NIR+Red+L)

Note: red, blue, green, and NIR indicate the reflectance in the red, blue, green, and near-infrared bands, respectively, and L is the optical soil conditioning factor, usually 0.5.

**Table 2:** Textural features and calculation formula.

Textural Index	Formula
Mean (mean)	$mean=\sum_{i,j=0}^{N-1}{iP}_{ij}$
Variance (var)	$var=\sum_{i,j=0}^{N-1}{iP}_{ij}{\left(i-mean\right)}^{2}$
Homogeneity (hom)	$hom=\sum_{i,j=0}^{N-1}i\frac{P_{ij}}{1+{\left(i-j\right)}^{2}}$
Contrast (con)	$con=\sum_{i,j=0}^{N-1}{iP}_{ij}{\left(i-j\right)}^{2}$
Dissimilarity (dis)	$dis=\sum_{i,j=0}^{N-1}{iP}_{ij}|i-j|$
Entropy (ent)	$ent=\sum_{i,j=0}^{N-1}{iP}_{ij}(-\ln P_{ij})$
Second Moment (sem)	$sem=\sum_{i,j=0}^{N-1}{iP}_{{ij}^{2}}$
Correlation (cor)	$cor=\sum_{i,j=0}^{N-1}{iP}_{ij}\left[\frac{\left(i-mean)(j-mean\right)}{\sqrt{{var}_{i}\times {var}_{j}}}\right]$

**Table 3:** Correlation coefficients determine the strength of correlation of variables.

Correlation Coefficient	Relevant Intensity
0.0–0.2	Very Weak Correlation or No Correlation
0.2–0.4	Weak Correlation
0.4–0.6	Moderate Correlation
0.6–0.8	Strong Correlation
0.8–1.0	High Correlation

**Table 4:** Textural features and SPAD correlation analysis.

Textural Feature	Wave Band				
	R	G	B	RE	NIR
mean	0.590 **	0.423 **	0.465 **	0.210	0.179
var	0.410 **	0.266 *	0.392 **	0.201	0.130
hom	−0.396 **	−0.282 *	−0.313 *	−0.188	−0.155
contrast	0.428 **	0.256 *	0.364 **	0.214	0.146
dis	0.431 **	0.281 *	0.362 **	0.179	0.151
entropy	0.373 **	0.257 *	0.271 *	0.186	0.142
sec	−0.333 **	−0.235	−0.247	−0.191	−0.139
corr	0.019	−0.052	0.066	0.075	−0.159

Note: * and ** indicate significant correlation at *p* < 0.05 and *p* < 0.01, respectively.

**Table 5:** Comparison of the prediction accuracy of different models.

Input Quantity	Model	Training Set			Validation Set		
		R^2^	RMSE	RPD	R^2^	RMSE	RPD
Vegetation Indices	XGBoost	0.812	0.921	2.184	0.749	1.432	2.071
	RF	0.713	1.443	1.862	0.673	1.501	1.442
	BPNN	0.680	1.512	1.483	0.634	1.682	1.236
	OIA	0.893	0.684	2.461	0.826	0.934	2.382
Textural Features	XGBoost	0.823	0.904	2.251	0.767	1.215	2.114
	RF	0.721	1.371	1.884	0.687	1.483	1.542
	BPNN	0.655	1.634	1.287	0.610	1.754	1.138
	OIA	0.865	0.802	2.364	0.816	0.893	2.320
Vegetation Indices + Textural Features	XGBoost	0.843	0.856	2.401	0.802	0.942	2.153
	RF	0.816	0.845	2.293	0.762	1.272	1.991
	BPNN	0.725	1.052	1.941	0.713	1.425	1.732
	OIA	0.931	0.564	2.782	0.877	0.675	2.674

## Data Availability

The data involved in the study can be obtained by contacting the authors.

## References

[1] Leschevin M., Ksas B., Baltenweck R., Hugueney P., Caffarri S., Havaux M. (2024). Photosystem rearrangements, photosynthetic efficiency, and plant growth in far red-enriched light. Plant J..

[2] Bauriegel E., Herppich W. (2014). Hyperspectral and chlorophyll fluorescence imaging for early detection of plant diseases, with special reference to fusarium spec. Infections on wheat. Agriculture.

[3] Dray F.A., Center T.D., Mattison E.D. (2012). In situ estimates of waterhyacinth leaf tissue nitrogen using a spad-502 chlorophyll meter. Aquat. Bot..

[4] Křížová K., Kadeřábek J., Novák V., Linda R., Kurešová G., Šařec P. (2022). Using a single-board computer as a low-cost instrument for spad value estimation through colour images and chlorophyll-related spectral indices. Ecol. Inform..

[5] Mukiibi A., Machakaire A.T.B., Franke A.C., Steyn J.M. (2024). A systematic review of vegetation indices for potato growth monitoring and tuber yield prediction from remote sensing. Potato Res..

[6] Du R., Lu J., Xiang Y., Zhang F., Chen J., Tang Z., Shi H., Wang X., Li W. (2024). Estimation of winter canola growth parameter from uav multi-angular spectral-texture information using stacking-based ensemble learning model. Comput. Electron. Agric..

[7] Kim K.Y., Haagenson R., Kansara P., Rajaram H., Lakshmi V. (2024). Augmenting daily modis lst with airs surface temperature retrievals to estimate ground temperature and permafrost extent in high mountain asia. Remote Sens. Environ..

[8] Kun X., Wei W., Sun Y., Wang Y., Xin Q. (2023). Mapping fine-spatial-resolution vegetation spring phenology from individual landsat images using a convolutional neural network. Int. J. Remote Sens..

[9] Chen A., Xu C., Zhang M., Guo J., Xing X., Yang D., Xu B., Yang X. (2024). Cross-scale mapping of above-ground biomass and shrub dominance by integrating uav and satellite data in temperate grassland. Remote Sens. Environ..

[10] Geng T., Yu H., Yuan X., Ma R., Li P. (2024). Research on segmentation method of maize seedling plant instances based on uav multispectral remote sensing images. Plants.

[11] Xiang H., Tian L. (2011). Development of a low-cost agricultural remote sensing system based on an autonomous unmanned aerial vehicle (uav). Biosyst. Eng..

[12] Jiang Y., Wei Z., Hu G. (2024). Detection of tea leaf blight in uav remote sensing images by integrating super-resolution and detection networks. Environ. Monit. Assess..

[13] Wang T., Gao M., Cao C., You J., Zhang X., Shen L. (2022). Winter wheat chlorophyll content retrieval based on machine learning using in situ hyperspectral data. Comput. Electron. Agric..

[14] Tian B., Yu H., Zhang S., Wang X., Yang L., Li J., Cui W., Wang Z., Lu L., Lan Y. (2024). Inversion of cotton soil and plant analytical development based on unmanned aerial vehicle multispectral imagery and mixed pixel decomposition. Agriculture.

[15] Schirrmann M., Giebel A., Gleiniger F., Pflanz M., Lentschke J., Dammer K.-H. (2016). Monitoring agronomic parameters of winter wheat crops with low-cost uav imagery. Remote Sens..

[16] Yuan Y., Wang X., Shi M., Wang P. (2022). Performance comparison of rgb and multispectral vegetation indices based on machine learning for estimating hopea hainanensis spad values under different shade conditions. Front. Plant Sci..

[17] Sun X., Yang Z., Su P., Wei K., Wang Z., Yang C., Wang C., Qin M., Xiao L., Yang W. (2023). Non-destructive monitoring of maize lai by fusing uav spectral and textural features. Front. Plant Sci..

[18] Khosravi I., Alavipanah S.K. (2019). A random forest-based framework for crop mapping using temporal, spectral, textural and polarimetric observations. Int. J. Remote Sens..

[19] Zheng H., Ma J., Zhou M., Li D., Yao X., Cao W., Zhu Y., Cheng T. (2020). Enhancing the nitrogen signals of rice canopies across critical growth stages through the integration of textural and spectral information from unmanned aerial vehicle (uav) multispectral imagery. Remote Sens..

[20] Li S., Yuan F., Ata-Ui-Karim S.T., Zheng H., Cheng T., Liu X., Tian Y., Zhu Y., Cao W., Cao Q. (2019). Combining color indices and textures of uav-based digital imagery for rice lai estimation. Remote Sens..

[21] Zhang W., Zhao L., Li Y., Shi J., Yan M., Ji Y. (2022). Forest above-ground biomass inversion using optical and sar images based on a multi-step feature optimized inversion model. Remote Sens..

[22] Xu W., Yang F., Ma G., Wu J., Wu J., Lan Y. (2023). Multiscale inversion of leaf area index in citrus tree by merging uav lidar with multispectral remote sensing data. Agronomy.

[23] Chen J., Wu S., Dong F., Li J., Zeng L., Tang J., Gu D. (2021). Mechanism underlying the shading-induced chlorophyll accumulation in tea leaves. Front. Plant Sci..

[24] Wu Z., Cui N., Zhang W., Gong D., Liu C., Liu Q., Zheng S., Wang Z., Zhao L., Yang Y. (2024). Inversion of large-scale citrus soil moisture using multi-temporal sentinel-1 and landsat-8 data. Agric. Water Manag..

[25] Pan K., Zhang X., Chen L. (2024). Research on the training and application methods of a lightweight agricultural domain-specific large language model supporting mandarin chinese and uyghur. Appl. Sci..

[26] Sivabalan K.R., Ramaraj E. (2020). Surface segmentation and environment change analysis using band ratio phenology index method—Supervised aspect. IET Image Process..

[27] Sotoodeh M., Moosavi M.R., Boostani R. (2019). A structural based feature extraction for detecting the relation of hidden substructures in coral reef images. Multimed. Tools Appl..

[28] Du R., Chen J., Xiang Y., Xiang R., Yang X., Wang T., He Y., Wu Y., Yin H., Zhang Z. (2024). Timely monitoring of soil water-salt dynamics within cropland by hybrid spectral unmixing and machine learning models. Int. Soil Water Conserv. Res..

[29] Elbasi E., Mostafa N., Zaki C., AlArnaout Z., Topcu A.E., Saker L. (2024). Optimizing agricultural data analysis techniques through ai-powered decision-making processes. Appl. Sci..

[30] Shi Y., Gao Y., Wang Y., Luo D., Chen S., Ding Z., Fan K. (2022). Using unmanned aerial vehicle-based multispectral image data to monitor the growth of intercropping crops in tea plantation. Front. Plant Sci..

[31] Bansal M., Malik S.K. (2020). A multi-faceted optimization scheduling framework based on the particle swarm optimization algorithm in cloud computing. Sustain. Comput. Inform. Syst..

[32] Munawar A.A., Zulfahrizal, Mörlein D. (2024). Prediction accuracy of near infrared spectroscopy coupled with adaptive machine learning methods for simultaneous determination of chlorogenic acid and caffeine on intact coffee beans. Case Stud. Chem. Environ. Eng..

[33] Yu X., Huo X., Qian L., Du Y., Liu D., Cao Q., Wang W.E., Hu X., Yang X., Fan S. (2024). Combining uav multispectral and thermal infrared data for maize growth parameter estimation. Agriculture.

[34] Wang R., Tuerxun N., Zheng J. (2024). Improved estimation of spad values in walnut leaves by combining spectral, texture, and structural information from uav-based multispectral image. Sci. Hortic..

[35] Evri M., Akiyama T., Kawamura K. (2008). Spectrum analysis of hyperspectral red edge position to predict rice biophysical parameters and grain weight. J. Jpn. Soc. Photogramm. Remote Sens..

[36] Kanke Y., Raun W., Solie J., Stone M., Taylor R. (2012). Red edge as a potential index for detecting differences in plant nitrogen status in winter wheat. J. Plant Nutr..

[37] Deng L., Mao Z., Li X., Hu Z., Duan F., Yan Y. (2018). Uav-based multispectral remote sensing for precision agriculture: A comparison between different cameras. ISPRS J. Photogramm. Remote Sens..

[38] Zheng Z., Yuan J., Yao W., Kwan P., Yao H., Liu Q., Guo L. (2024). Fusion of uav-acquired visible images and multispectral data by applying machine-learning methods in crop classification. Agronomy.

[39] Dhakal R., Maimaitijiang M., Chang J., Caffe M. (2023). Utilizing spectral, structural and textural features for estimating oat above-ground biomass using uav-based multispectral data and machine learning. Sensors.

[40] Wu J., Bai T., Li X. (2024). Inverting chlorophyll content in jujube leaves using a back-propagation neural network–random forest–ridge regression algorithm with combined hyperspectral data and image color channels. Agronomy.

[41] Peng Y., Nguy-Robertson A., Arkebauer T., Gitelson A. (2017). Assessment of canopy chlorophyll content retrieval in maize and soybean: Implications of hysteresis on the development of generic algorithms. Remote Sens..

